# Separation and Analysis of Connected, Micrometer-Sized, High-Frequency Damage on Glass Plates due to Laser-Accelerated Material Fragments in Vacuum

**DOI:** 10.3390/jimaging10050101

**Published:** 2024-04-26

**Authors:** Sabrina Pietzsch, Sebastian Wollny, Paul Grimm

**Affiliations:** 1 Faculty of Media, Darmstadt University of Applied Sciences, Schöfferstraße 3, 64295 Darmstadt, Germany; 2 Information Center for Education, DIPF|Leibniz Institute for Research and Information in Education, Rostocker Straße 6, 60323 Frankfurt am Main, Germany; 3 Institute for Computer Science, Goethe University, Norbert-Wollheim-Platz 1, 60323 Frankfurt, Germany

**Keywords:** impact, glass plate, surface, laser–matter interactions, image processing, object separation, recycling process optimization

## Abstract

In this paper, we present a new processing method, called MOSES—Impacts, for the detection of micrometer-sized damage on glass plate surfaces. It extends existing methods by a separation of damaged areas, called impacts, to support state-of-the-art recycling systems in optimizing their parameters. These recycling systems are used to repair process-related damages on glass plate surfaces, caused by accelerated material fragments, which arise during a laser–matter interaction in a vacuum. Due to a high number of impacts, the presented MOSES—Impacts algorithm focuses on the separation of connected impacts in two-dimensional images. This separation is crucial for the extraction of relevant features such as centers of gravity and radii of impacts, which are used as recycling parameters. The results show that the MOSES—Impacts algorithm effectively separates impacts, achieves a mean agreement with human users of (82.0 ± 2.0)%, and improves the recycling of glass plate surfaces by identifying around 7% of glass plate surface area as being not in need of repair compared to existing methods.

## 1. Introduction

**Figure 1:**
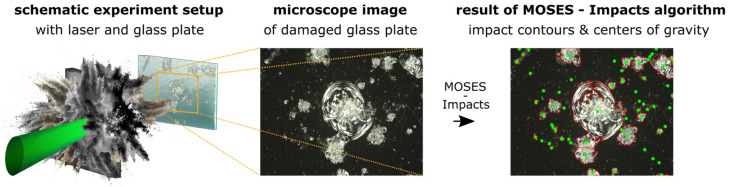
Examination sequence of damage on glass plates resulted from laser–matter experiments, which are used to protect sensitive diagnostics. (**Left**): Schematic of the experimental setup; a laser beam (green) is focused on an object (gray), which is destroyed. During destruction, material fragments are accelerated and hit a glass plate behind the object. (**Center**): An enlarged post-experimental image of the damaged glass plate. (**Right**): In this case, the resulting image shows contours in red and the centers of gravity in green. In addition, connected impacts are separated and marked as individual damages.

Damage to glass plate surfaces in laser–matter experiments (Figure 1 (left and center)) causes unwanted products that increase experimental costs and limit the number of experiments that can be carried out per experiment period.

In these laser–matter experiments, a high-intensity laser beam is directed to an object in a vacuum, called a target (Figure 1 (left)). The high laser energy involved causes the target to explode and the released material fragments to reach a relative speed of several kilometers per second [1]. In this context, the laser energy, the target material, and the size of the accelerated material fragments are decisive for the degree of damage to a glass plate. As a result, small fragments, called debris, mainly settle as a thin film of material on surrounding surfaces, while larger fragments, called shrapnels, permanently limit the lifetime of the experimental apparatus due to impacts [2].

The study of laser-induced damage is of increasing interest as high-power laser systems, such as the Center for Pulsed Lasers (CLPU) in Spain or the Extreme Light Infrastructure (ELI) beamlines facility in the Czech Republic with high-repetition rates of 10 Hz [3], are currently being commissioned. These high-power, high-repetition lasers, on the one hand, enable scientists to research basic physics experiments faster than former laser systems. On the other hand, the facilities are faced with an increased factor of wear and tear [4]. In particular, damage to optical components is an increasing problem for highly frequented experimental operations [5]. To minimize costs and extend the lifetime of optical components, inexpensive glass protection plates are used to protect expensive optical components from direct damage [6].

Camera-based recycling processes [7] that help to minimize the optical effects of damaged areas, caused by impacts on optical components, have been developed. A state-of-the-art recycling method is surface smoothing through a small carbon-dioxide laser (CO_2_) laser system, which is used at the largest laser system in the USA, the National Ignition Facility (NIF) [7]. Surface smoothing transforms impacts into smooth and harmless cone-shaped dips [7]. For such recycling procedures, the precise location of impacts [8], as well as the optimal laser spot size are of interest.

Due to the high frequency of the impacts and a micrometer-sized structure, impacts may also be clustered and connected. Such connected impacts are potentially recognized as a single damage and thus classified as major damage, rather than multiple smaller damages. This circumstance may lead to an oversized spot size of the CO_2_ recycling laser, which implies that undamaged areas are unnecessarily included in the recycling process. A separation of connected impacts in the recognition of damages is therefore crucial for further optimizing the recycling process.

In this work, a processing method for microscope images of damaged glass plates has been developed to minimize the recycling laser spot size error in recycling processes. The aim of the processing method is to separate connected damages based on the microscope glass plate images by combining multiple image processing functions. The method consists of a two-step process and is structured as follows: first, the Pre-Processing Phase, where impacts are detected, and second the Processing Phase, where connected impacts are separated (Figure 1 (right)). To lead our research on the recognition and evaluation of shrapnel impacts on glass plates, two research questions were defined:

RQ1: What is the effect of the damage separation on the total area to be repaired?

RQ2: What is the similarity of the developed processing method in detecting impacts on glass plates compared to human users?

The main contribution of this paper is a new method for detecting laser–matter impacts that allows an optimization of recycling processes of damaged glass plates through separating impacts. This method differs from existing detection methods and algorithms in the field of quality glass inspection [9] by detecting a large number of impacts on small areas with connected impacts instead of a single impact on larger areas. The second contribution is the evaluation of similarity of detection results compared to human users.

The following sections are structured as follows: Section 2 provides an overview of related work on the topic, while Section 3 presents the experiment information and the digitization of the glass plates. In Section 4, a definition of a shrapnel impact is given, and, in Section 5, the processing method is presented with a flow chart. In Section 6, the method is evaluated in comparison to a human user study and research results are discussed in relation to the research questions. Finally, in Section 7 future work is presented.

## 2. Related Work

In this section, we want to give an overview of related work in the field of damage detections on surfaces and an analysis of accelerated material fragments by grouping findings into two major topics: first are the results of accelerated material fragments, caused by laser–matter interaction as well as recycling methods to minimize damage; and second is the state of the art in the computer vision of glass plate inspections.

In 1998, Lebert et al. [10] provided the first description of surface damages caused by laser–matter interactions in a vacuum with different target materials, while four years later, in 2002, Azuma et al. [11] determined the average area of impacts via the angular orientation of the glass plate to the target normal and showed that the average impact size remains small from an angle of 60°. In 2005, Higashiguchi et al. [12] investigated the area of damages by their frequencies and thus provided the insight that impacts with a size between 3 and 10 µm occur most frequently. Three years later, Andrew et al. [2] observed that the angle of incidence of the shrapnel on a glass plate determines the shape of the impact. Shrapnel with small angles of incidence produces circular impacts and becomes comet-like as the angle increases [2]. Furthermore, Andrew et al. [2] documented that the most energetic material fragments gather in the center of the impact area and the distribution of impacts decreases symmetrically around the center.

In 2013, Martinkova et al. [13] extended the previous two-dimensional results with the volume of individual impacts. Plasticine was used to create impressions of the impacts of the damaged surfaces. This enabled them to estimate the volume of material removed from a glass plate. For impacts with a diameter of (1.0–2.0) mm, the volume was (0.14–0.48) mm^3^ and for larger diameters between (2.75–8.4) mm, the volume was (0.45–37.4) mm^3^ [13]. Furthermore, correlations could be found between the impact diameter, the impact depth, the shrapnel diameter, and its material density. These correlations enabled them to estimate the shrapnel velocity, which resulted in (288–534) m/s for the small impact diameters [13]. In addition to the previous experimental results, Aubert et al. [14] provided a contribution to the topic of impact simulations in 2019. Their simulation of impacts differed from previous impact studies due to the point of origin. The simulated impacts refer to the location on the target surface where the laser–matter interaction takes place. For these investigations, a finitely thick target is assumed, which is non-explosive. Damage in the form of larger craters occurs on the target surface due to the interaction. The simulation enables the prediction of crater dimensions as a function of laser energy. Bude et al. [5], in this context, highlight that all high-power laser systems have similar sources of damage.

In the US, the National Ignition Facility (NIF) is equipped with the largest high-power laser system. Its optics have thousands of damages greater than 10 µm, as well as many more damages smaller than 10 µm in size. Tobin et al. [15] proposed guidelines for conducting experiments at the NIF to increase the lifetime of protective glass plates against shrapnel damage. The authors discovered a correlation between shrapnel size, shrapnel velocity, and the thickness of a glass plate required to withstand a collision. Tobin et al. suggested that a 50 µm shrapnel with a velocity of 1 km/s requires a 100 µm thick glass plate to withstand, while a shrapnel four times as large and twice as fast would require a 1 mm thick glass plate. Tobin et al. [15] also referred to the potential danger of breaking protective glasses, which may result in higher material loss than through impacts. They explained that the spallation typically generates a cone-shaped impact [15]. Typical quantitative measurements of shrapnel size can be characterized with as aerogels that absorb shrapnels [1], shadowgraphs [16], and high-speed video recordings [17]. The use of aerogels stops shrapnels before touching the surface and is solely suitable for measuring the shape and volume of shrapnels. It cannot be used to draw conclusions about the potential damage caused. Shadowgraphs and high-speed video recordings do not interact with the shrapnel. However, information about shrapnel volume has to be extrapolated from two-dimensional images.

Spaeth et al. [7] emphasized the importance of developing efficient strategies to reduce and fix damage to glass optics. One promising strategy is the optics recycling loop, which involves steps designed to evaluate, repair, and minimize damage to optics [7]. These steps include regular replacement, cleaning, polishing, coating, and optics testing [7]. Carr et al. [18] presented comprehensive strategies for mitigating, managing, and repairing the damage that occurs at NIF, which is crucial in preserving the functionality of the optics. The damage is mainly caused by laser-induced processes. The recycling process involves using automated CO_2_ laser systems that apply Rapid Ablation Mitigation (RAM) to repair the damaged optics. RAM uses concentrated laser beams to remove damaged areas and repair optics [18]. The recycling process is initiated by measuring the surface of optics on an automated inspection microscope, which locates all damages larger than about 7 µm [18]. Afterwards, the automated CO_2_ laser systems are used to repair any damaged area larger than 10 µm [18]. Miller et al. [19] consider, in a similar process, previously repaired optics. Their examined optical surface of 43 cm × 43 cm is scanned and analyzed by the recycling process within 4 h by a resolution of about 5 µm per pixel [19]. In this process, the previously repaired spots have to be identified first. Thereafter, new damages can be classified and repaired accordingly [19]. Negres et al. [8] rely on human evaluations for analyzing damage, which is a labor intensive process. This procedure, however, prevents possible threshold errors in image processing steps. In their work, they stress the importance of detailed knowledge on the locations of damages to design more robust recycling processes in the future [8].

Research in this area shows a clear trend for the development of more advanced diagnostic and repair technologies that focus more on automated systems. The constant improvement in these technologies is essential for the stability and lifetime of high-energy laser systems.

Computer Vision enables non-contact inspection systems based on image processing algorithms that are used to detect surface damages automatically [9,20,21,22,23]. For example, Peng et al. [9] provide an overview of improving glass quality at production by detecting defects. Zhao et al. [20] and Liu et al. [21] make use of computer vision and developed glass defect classification methods that are useful for low and differing resolutions. While Makaremi et al. [22] described a new approach in 2018 for texture defect detection based on a modified version of the local binary pattern (LBP), Hoang et al. [23] presented a method for detecting spall objects using Gabor filters, texture analysis, and adaptive optimized logistic regression models. These studies contributed to the improvement in defect detection and classification techniques, applying advanced image processing methods to increase product quality in various industrial applications and to improve safety in the construction industry.

By analyzing existing computer vision processes for detecting shrapnel impacts on glass plates, an overarching structure can be identified in the inspection systems presented: The majority of image processing algorithms follow a typical pattern. First, the removal of background information is carried out by applying filter operations [9], followed by a segmentation method [23] or edge detection method, used to identify impact contours [20]. Finally, binarization of the images is undertaken [9,20,22,23].

## 3. Experiment Parameter and Data Acquisition

**Figure 2:**
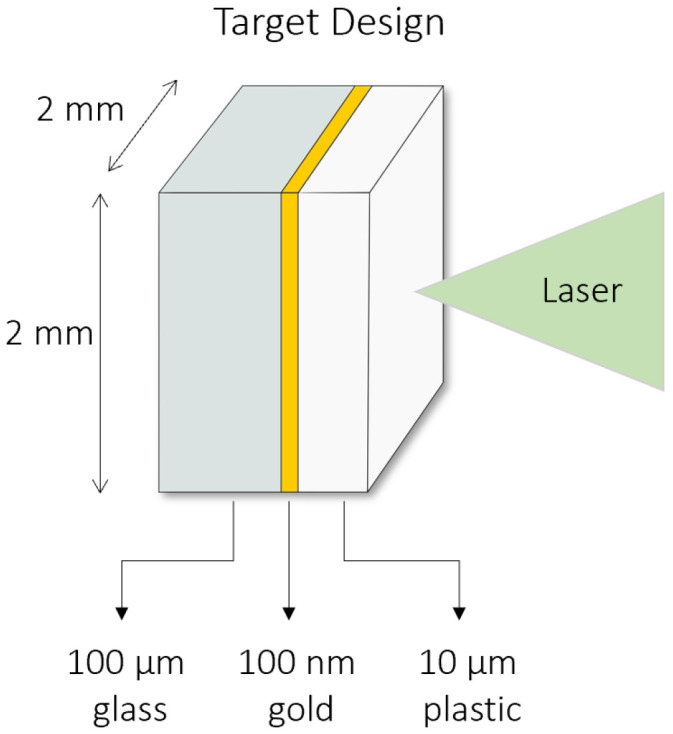
Schematic drawing of the layered target interacting with the laser. The target is made of three different materials: plastic, gold, and glass.

This chapter provides the key data for damaged glass plates to be investigated in this work. The experimental setup, which is shown in Figure 1 (left), was carried out as part of an experimental campaign at the Prague Asterix Laser (PALS) in the Czech Republic. The target consisted of plastic (layer thickness: 10 µm), gold (layer thickness: 100 nm), and glass (layer thickness: 100 µm), with a surface area of 2 mm × 2 mm (see Figure 2).

**Figure 3:**
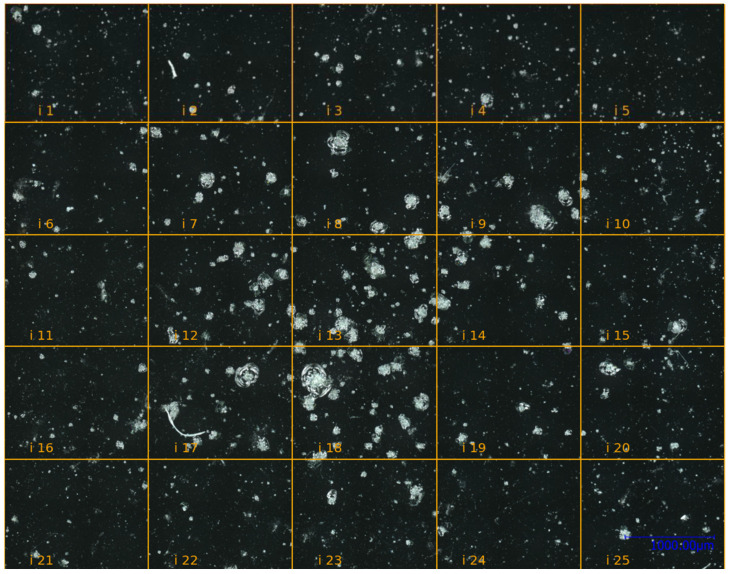
Microscope image of a heavily damaged area on glass plate no. 41 with separated sections. The 25 image sections (**i1**–**i25**) were combined into a total image of 15,235 px × 11,883 px.

Glass plates were placed 522 mm behind targets and served as protection for sensitive diagnostics in the vacuum chamber. During this campaign, 19 glass plates showed damage from shrapnel impacts. The size of shrapnels could not be determined due to limited space in the experimental setup. For the work presented here, glass plate no. 41 is primarily used and serves as a proxy for glass plates that are highly frequented by shrapnel damages. Its dimensions are 76 mm × 52 mm. The glass plate comprises a heavily damaged area (Figure 3), which covers about a quarter of the glass plate. The damage was caused by a laser–matter interaction through a laser single shot with a duration of 250 ps and a pulse energy of 151 J. The diameter of the laser focus on the target surface was 400 µm.

**Figure 4:**
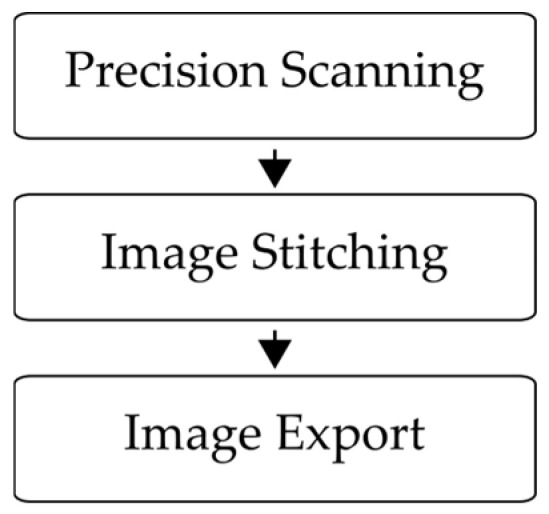
Block diagram of the image acquisition process.

In Figure 4, a block diagram is presented to visualize the image acquisition process. The glass plate is scanned with a digital microscope in a high-resolution precision scanning process with 10 × 10 individual images at a resolution of 0.5 µm per pixel (px) (Precision Scanning). Thereafter, the 10 × 10 images are combined to a total image of 15,235 px × 11,883 px (Image Stitching). This resulting image is then exported and saved for further processing (Image Export).

## 4. Definition of a Shrapnel Impact

For determining the accuracy of shrapnel impact recognition on glass plate surfaces, a definition of a shrapnel impact is needed. Since there is no general definition in the literature so far that describes shrapnel impacts in detail, we introduce and use a definition in the following part, which considers borderline cases, on the one hand, and distinguishes areas of impact, on the other hand.

A shrapnel impact on a glass plate is a material change caused by accelerated fragments of a material, which hit the glass surface. The impact area of a shrapnel can be divided into four subcategories, which can be described as follows:

(a) Crater area;
(b) Ring area;
(c) Partially disconnected material area;
(d) Molten material area.

**Figure 5:**
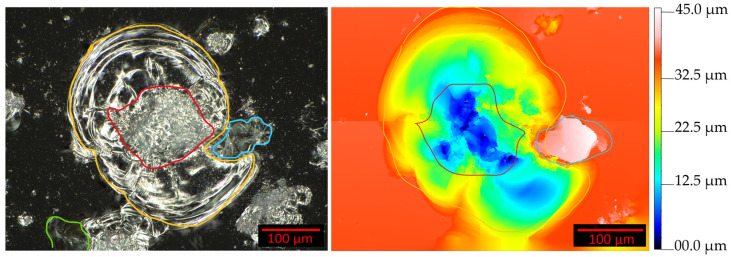
Definition of an impact. The impact area is divided into four subcategories: (a) crater area (red); (b) ring area (yellow); (c) partially disconnected material area (green); (d) molten material area (blue). (**Left**): Two-dimensional image. (**Right**): Three-dimensional image as a color map.

Figure 5 shows two images of the same impact, captured with two different microscopes and divided into four subcategories, drawn as colored contours. The left image shows a two-dimensional image, acquired with a digital microscope. The right image shows a corresponding three-dimensional image as a color map, acquired with a confocal microscope.

Both categories—the crater (Figure 5, red contour) and ring areas (Figure 5, yellow contour)—are regions that lost material compared to an undamaged glass plate surface. They usually differ in the depth of the damage and constitute large parts of the impact areas. The three-dimensional image helps us to detect the molten material (Figure 5, blue contour). It can be detected through a deviation in height relative to the undamaged glass plate surface and hardly shows any color changes in a two-dimensional image.

**Figure 6:**
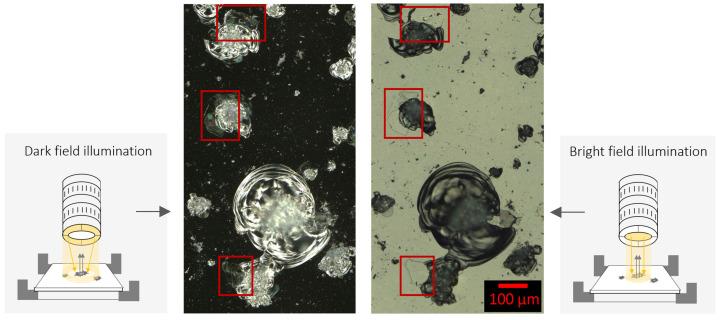
For the visibility of category c—partially disconnected material—two identical image sections were taken with different illumination settings. Larger differences between the illumination settings are highlighted with red boxes. (**Left**): Dark field. (**Right**): Bright field illumination.

A dark field illumination setting enables the detection of partially disconnected material (Figure 5, green contour) by indicating spectral color components in these areas, while a bright field illumination setting shows deviations from the background color (Figure 6, red boxes).

## 5. Processing Method

In this section, we describe the proposed processing method, the MOSES—Impacts (Method for Outlining and Separating Existing Surface—Impacts) algorithm, for detecting and separating connected shrapnel impacts on glass plate surfaces, as well as calculating the centers of gravity of the impacts. To detect and separate shrapnel impacts (as foreground) from intact glass plate areas (as background), the identification of impact contours is a crucial step. This method focuses on accurately identifying the impact area without dividing it into sub-areas and labeling them.

**Figure 7:**
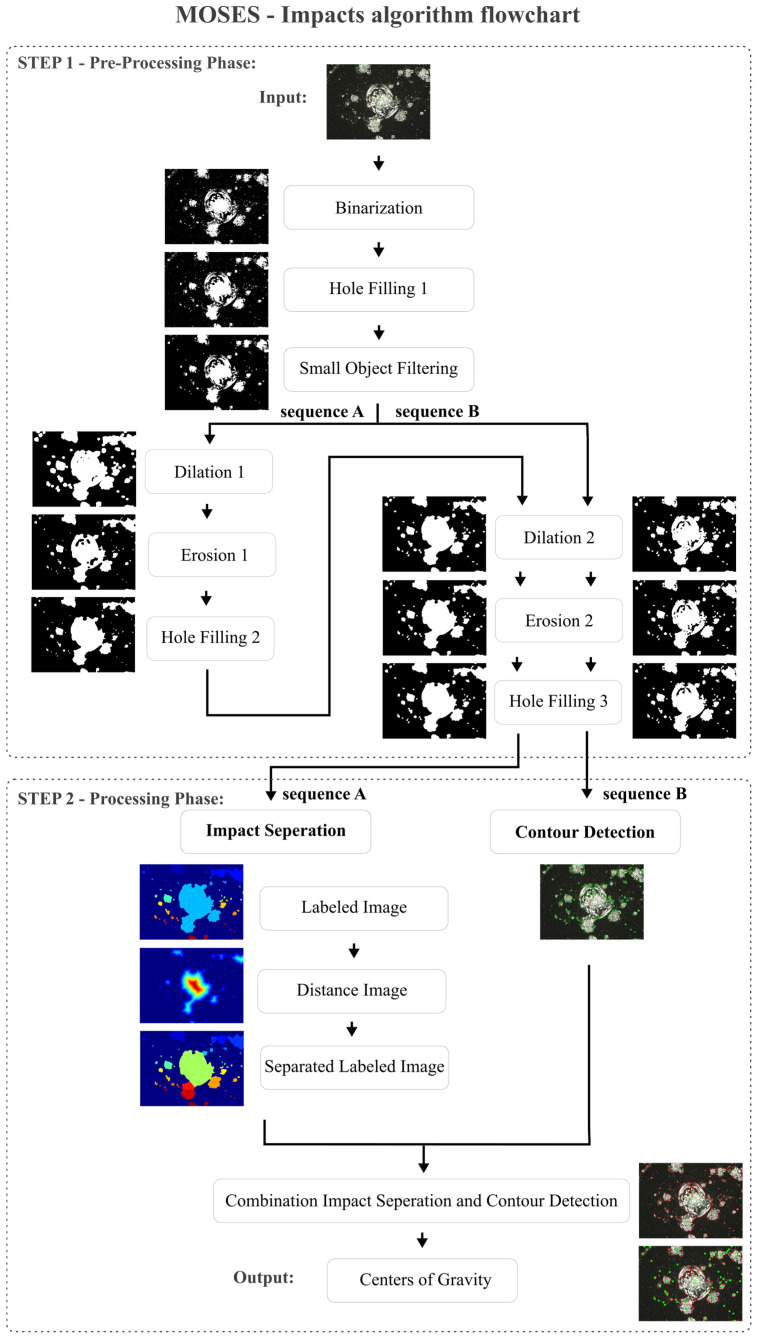
MOSES—Impacts algorithm flowchart of the 2-step processing method for separation and detection impacts on glass surfaces.

The MOSES—Impacts algorithm is shown as flow chart in Figure 7, which was implemented in Python 3.9.8, including the open-source libraries of cv2, numpy, matplotlib, math, sys, pandas, PIL, skiamge, scipy, argparse, and imutils. It utilizes color images as input data and consists of two steps: The Pre-Processing and Processing Phase. Both steps are described in detail in Section 5.1 and Section 5.2.

**Figure 8:**
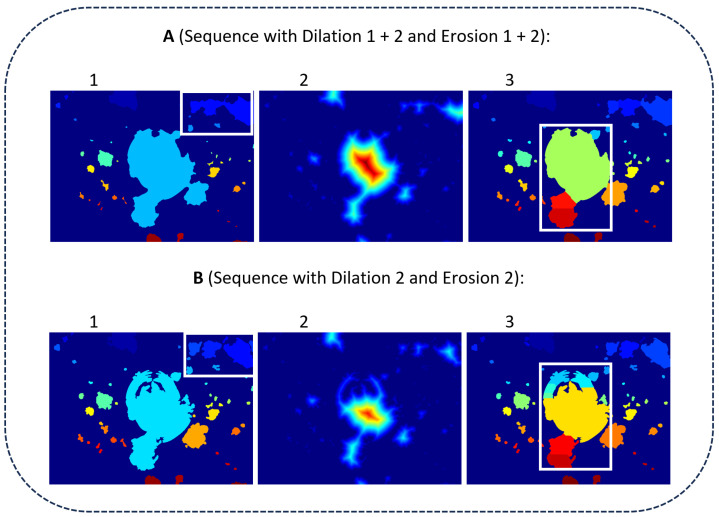
Intermediate labeled images of the MOSES–Impacts algorithm after Pre-Processing Phase. Sequence A achieves better separation.

A characteristic of the MOSES—Impacts algorithm is the parallel sequence, which starts from the Pre-Processing Phase and extends to the Processing Phase. In this parallel sequence, which is divided into sequence A and sequence B, two different images are calculated: one for the separation and one for contour calculation. This is required to obtain the best possible result between impact separation and contour detection. The difference between sequence A and sequence B at the end of the Pre-Processing Phase in combination with the Impact Separation step is shown in Figure 8. Here, the applied Dilation 1 + 2 and Erosion 1 + 2 in Figure 8(A1) result in a single recognized impact (white box in the upper right corner). In contrast, Dilation 2 and Erosion 2 applied in Figure 8(B1), without Dilation 1 and Erosion 1, result in multiple separated impacts (white box in the upper right corner). In Figure 8(A2,B2), the distance images of detected impacts of Figure 8(A1,B1) are shown. The distance image of sequence A, in general, shows larger distances compared to the distance image of sequence B. In Figure 8(A3,B3), the separation of connected impacts is visualized. Compared to sequence A, sequence B tends to separate individual impacts as well (white center boxes).

To assess these results, three measures should be used: 1. the number of detected impacts; 2. the accuracy of contour detection; and 3. the performance in separating connected impacts. Regarding the first two measures, Sequence B leads to more accurate results. However, the performance in separating impacts of sequence B is surpassed by sequence A. In order to utilize the advantages of Sequence A and Sequence B, both sequences are applied and combined at the end of the Processing Phase. Both phases are described below and are structured as follows:

In step 1—Pre-Processing Phase—a color image is converted into a greyscale image and binarized (Binarization) (see Figure 7). Existing holes in the foreground are then filled (Hole Filling 1) and a filter is applied afterwards. The filter removes small impacts (Small Object Filtering), as the resolution of these is too low meaning they could not be separated from image and digitization noise. This image is further processed in sequence A with two morphological operators (Dilation 1 + 2 and Erosion 1 + 2) and in sequence B with only one morphological operator (Dilation 2 and Erosion 2), resulting in two different images being generated. The main difference between Dilation 1 and Dilation 2 in this parallel sequence is the number of single operator repetitions. In Dilation 1, the image is dilated 10 times, while in Dilation 2, it is dilated 4 times. Similarly, in Erosion 1, the image is eroded ten times, while in Erosion 2, it is eroded once. After each erosion, existing holes in the foreground are filled again (Hole Filling 2 and 3) to ensure that a larger impact can be recognized as such.

In step 2—Processing Phase—the two resulting images of Hole Filling 3 are further processed in sequence A and sequence B. In sequence A, connected impacts can automatically be separated from each other (Impact Separation). For the separation, a labeling (Labeled Image) is used at first, then a distance calculation of foreground to background pixels (Distance Image), which is supplemented by a watershed transformation, is carried out and finally a new labeled image (Separated Labeled Image) is calculated. In sequence B, the contours of impacts are calculated with a contour tracking code (Contour Detection). The images of sequence A and sequence B are then merged to create a combined image of separated impacts and optimized contours (Combination Impact Separation and Contour Detection). The combined image is created by adding the separation lines of the Separated Labeled Image step to the Contour Detection image. In this context, contours identified in the Pre-Processing Phase serve as a hard boundary between the foreground and background and are used for the calculation of the centers of gravity for each impact (Centers of Gravity).

### 5.1. Step 1—Pre-Processing Phase

This section describes the Pre-Processing Phase mathematically. It is introduced by binarization. In general, binarization in combination with a correct threshold leads to a good extraction of an impact in an image. In this case, an automatically determined threshold value *t* is calculated by Nobuyuki’s OTSU method [24], which delivers usable results even with slight variations in image brightness. The OTSU method used is defined by the following scheme [25]:

(1) $$\sigma_{in}^{2}\left(t\right)=\omega_{1}\left(t\right)\sigma_{1}^{2}\left(t\right)+\omega_{2}\left(t\right)\sigma_{2}^{2}\left(t\right)$$

where ω_1_(*t*) and ω_2_(*t*) are the probabilities that a pixel is identified as foreground or background, while $\sigma_{1}^{2}$(*t*) and $\sigma_{2}^{2}$(*t*) are the variances in the pixel intensities in the foreground and background. After applying the thresholding method, a binary image

(2) $$Bin(x,y)=\left\{\begin{array}{l} 0,\;background\;color\\ 1,\;forderground\;color \end{array}\right.$$

consists of foreground and background areas incorporating holes, which are then filled through a modified flood fill algorithm. The flood fill algorithm is based on matching neighboring pixels by changing each background pixel found in a hole to a foreground pixel [26]. The modified flood fill algorithm consists of a recursive approach instead of an iterative approach, which stores colored neighbor pixels by a stack [27]. In this way, no pixel is checked and filled multiple times, which saves computing time. The steps of the modified flood fill algorithm are described in Algorithm 1.
**Algorithm** **1.** The steps of the modified flood fill.Initialization—choosing a background pixel $p_{0}$= $\left(x_{0},y_{0}\right)$ of the image Bin(x,y) as the starting point and creating a foreground color-filled pixel mask Mask(x,y)=1 in the same shape as the image.Creating an empty stack and adding the starting point $p_{0}$ onto the stack.Iterating while the stack is not empty:a.Storing the first element of the stack as $p_{n}$ = $\left(x_{n},y_{n}\right)$;b.Removing the top element from the stack;c.Checking if $p_{n}$ is within the image boundaries and has a background color;d.**If yes:**i.Setting $Mask(x_{n},y_{n})=0$.ii.Adding the four neighbor pixels to the stack:$$\begin{array}{c} p_{top}\left(x_{n},y_{n-1}\right)\\ p_{bottom}\left(x_{n},y_{n+1}\right)\\ p_{right}\left(x_{n+1},y_{n}\right)\\ p_{left}\left(x_{n-1},y_{n}\right) \end{array}$$Setting Bin(x,y)=Mask(x,y)—**end**.

After holes are filled, contours of impacts are tracked. The retrieval method from Suzuki and Abe [28] is used for contour tracking. This method identifies contour points of an object by searching for a foreground pixel with a value of 1 first. Once found, the algorithm checks the 8 neighboring pixels counterclockwise and follows object pixels until the edge of an object is reached. At this point, the contour pixel is stored and the retrieval method continues checking neighboring pixels, follows edge pixels found counterclockwise, and stores them in a list. The algorithm stops when it reaches the first edge pixel again. All the edge pixels found correspond to the contour points that form a polygon and represent the object’s contour. This process is repeated recursively for all object pixels found until there are no more object pixels left to be examined. The results of the retrieval method are used to determine the impact area.

Subsequently, small impacts with an area lower than 100 px, approximately corresponding to an impact with an 6 µm diameter are removed from the contour due to a low image resolution and the limitation of recycling methods, which are, in general, limited to spot sizes larger than 10 µm [18].

**Figure 9:**
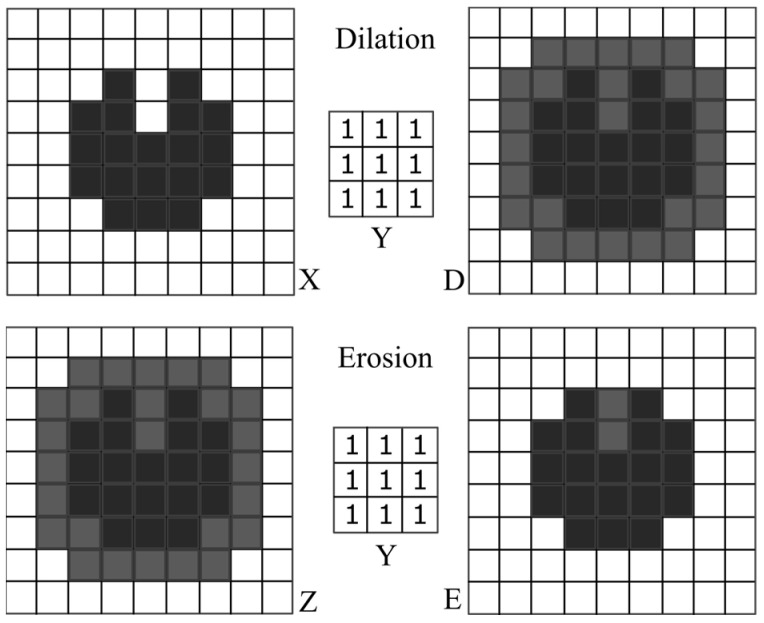
Illustration of morphological operator dilation and erosion. (**Top Left**): Original image X, foreground pixel value = 1, background pixel value = 0. (**Top Right**): Dilated image D by a 3 × 3 square structural element Y, shown in grey with the foreground area added to it. (**Bottom Left**): Dilated image Z for erosion, both foreground pixel values, black and grey = 1, and background pixel value = 0. (**Bottom Right**): Eroded image E by a 3 × 3 square structural element Y, shown in grey, and the remaining foreground pixels are shown in black.

From here, two different images are created, which continue being processed in sequences A and B. Dilation D and Erosion E belong to the morphological operators and optimize the shape of the impacts (Figure 9).

With both operators, a structural element *Y* is moved over the image *X*(*x*,*y*) or *Z*(*x*,*y*) [29]. The size of structural element *Y* should be in relation to the shape of the image to be processed. In traditional image processing, the size of the structural element *Y* and the number of repetitions for filters are usually determined experimentally to find the best configuration. A smaller structural element *Y* (e.g., 3 × 3 or 5 × 5) is suitable for small details or fine structures. For larger objects or areas, on the other hand, a larger structural element *Y* is recommended (e.g., 7 × 7 or 9 × 9). Most structural elements *Y* are square (e.g., 3 × 3, 5 × 5, 7 × 7), but asymmetrical structural elements *Y* (e.g., 3 × 5) are also possible. The dilation is based on the Minkowski addition:

(3) X⊕Y=D

The image *D*(*x*,*y*) is therefore a union of the set *Y* of all displacements from the structural element *Y* + *x*, where the translation vectors *x* come from the image *X*(*x*,*y*). The erosion is calculated according to the Minkowski subtraction:

(4) Z⊖Y=E

It results in the foreground picture *Z*(*x*,*y*) being reduced again. In this context, the subtraction is not an inversion of the addition, as illustrated in Figure 9; here, *X*(*x*,*y*) ≠ *E*(*x*,*y*).

### 5.2. Step 2—Processing Phase

This section describes the Processing Phase mathematically, which receives images with filled holes from the Pre-Processing Phase in sequences A and B. The resulting images can be used for the separation of connected components or contours. The contours are calculated using “Suzuki’s and Abe’s approach” [28] again, which has already been described in Section 5.1. The separation of connected impacts is reached through a combination of distance images D*(*x*,*y*) and a watershed transformation. For the calculation of the distance image D*(*x*,*y*), the Euclidean distance transformation is used, which calculates the smallest Euclidean distance d to the background pixel b within its 8 neighboring pixels and assigns it to each foreground pixel f [30].

(5) $$\|d\|=\sqrt{{\left(b_{x}-f_{x}\right)}^{2}+{\left(b_{y}-f_{y}\right)}^{2}}$$

To separate the impacts, Beucher’s [31] watershed transformation is applied to the distance image D*(*x*,*y*), shown in false color in Figure 7. It interprets the Euclidean distances d in the distance image D*(*x*,*y*) and can be symbolically regarded as a “mountain landscape” [30], whereas the distance value d indicates the height of the mountain range. The watershed transformation then symbolically floods the valleys (local minima) with water, starting from the minimum [30,32,33]. When two filled valleys meet, a “dam” is built. This dam is called the watershed and acts as an impact boundary. As soon as the entire image is filled, the algorithm ends [30] and the resulting impacts are separated from each other.

## 6. Evaluation and Discussion

This chapter presents an evaluation of the proposed MOSES—Impacts algorithm based on the research questions. For evaluating both research questions (RQ1, RQ2), we randomly selected image sections presented in Figure 3, which are used to evaluate the algorithm. This serves to limit the cognitive load of participants. The image sections selected are referred to as “i3”, “i12”, “i14”, and “i22” in Figure 3.

### 6.1. The Effect of Damage Separation

**Figure 10:**
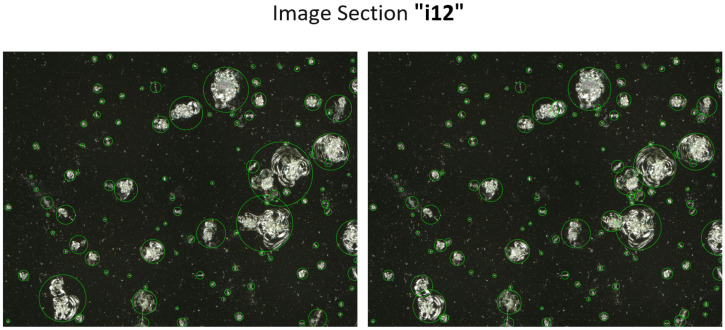
Recycling laser spot radii of detected impacts—(**Left**): Before a separation. (**Right**): After a separation.

This chapter discusses the influence of the separation of connected impacts on the recycling process. For this purpose, a circular bounding box was placed around each impact center of gravity. The required radius for the laser spot recycling of each impact was determined by the minimum radius that covered the whole object contour. This calculation was carried out with the resulting image of sequence B (no separation), as well as with the combined image of sequence A and B (including separation). Figure 10 exemplarily shows image “i12”, with circular bounding box areas marked in a green color that represent the identified areas for a recycling procedure. While the left image shows the selected areas without a separation of connected impacts, the right image shows the selected areas with a separation of connected impacts. In order to calculate the “saved area of undamaged glass”, two binary images of filled circular bounding boxes were subtracted from each other. The resulting difference image identifies areas differing between both images, whereas non-zero pixels are counted. To obtain the relative “saved area of undamaged glass”, the number of non-zero pixels is divided by the total image section area.

The result for image “i12” indicates that 14.84% of undamaged glass plate area could be saved in a recycling process with the separation of impacts. Furthermore, in image “i3”, 3.27%, in “i14”, 6.37%, and in “i22”, 3.57% could be saved through the separation step. On average, around 7% could be saved through impact separation. This shows that the separation of impacts is necessary to make the recycling process more efficient.

### 6.2. Similarity of the MOSES—Impacts Algorithm Compared to Human Users

A user study was carried out to evaluate the similarity of the method compared to six human users. The users were given image sections “i3”, “i12”, “i14”, and “i22” in Figure 3, preprocessed up to the Small Object Filtering step. For this purpose, users were advised to manually add the centers of gravity of impacts to the image sections, which resulted in 24 labeled image sections as a baseline for the comparison. The definition of an impact in Figure 5 was shown and explained to the users in advance.

**Figure 11:**
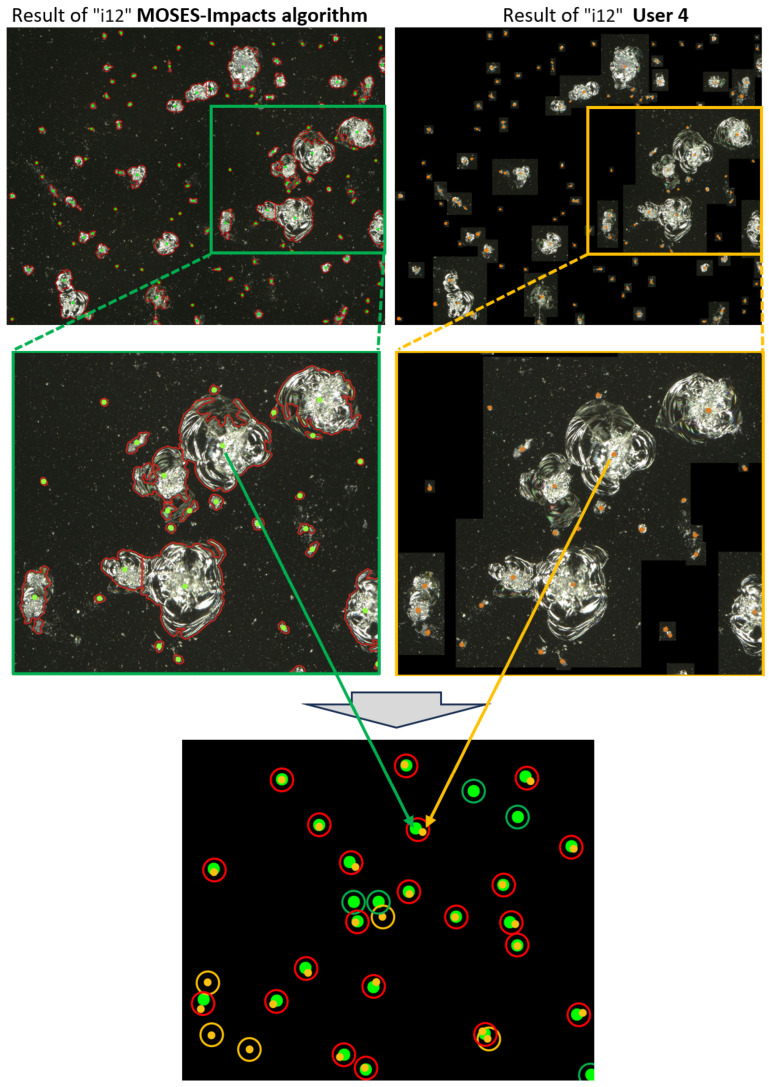
Result image of image section “i12” and the corresponding centers of gravity. (**Top Left and Center Left image**): Centers of Gravity marked with MOSES—Impacts algorithm. (**Top Right and Center Right image**): Centers of Gravity marked manually by human user 4. (**Center Left and Center Right image**): A detailed view of image section “i12” with centers of gravity, marked by a green / yellow box in the images above. (**Bottom image**): A fused image of centers of gravity from MOSES—Impacts Algorithm, as well as user 4. Red circles are drawn, if both detected an impact; green circles are drawn, if the MOSES—Impacts algorithm detected an impact solely; orange circles are drawn, if the user detected an impact solely.

An exemplary labeling result of user 4 is shown in Figure 11. On the left side, the centers of gravity by the MOSES—Impacts algorithm are marked in green, while on the right side, the participants identified centers of gravity, marked in orange.

The Jaccard index is used to evaluate the image by comparing the centers of gravity of the MOSES—Impacts algorithm and the impacts manually identified by the users. The Jaccard index is a measure of the similarity of sets *M* and *U*. It is often referred to as IoU (Intersection over Union) [23] and is an established index in the evaluation of semantic segmentation models [24]. It is defined as follows:

(6) $$J(M,U)=\frac{|M\cap U|}{|M\cup U|}$$

**Figure 12:**
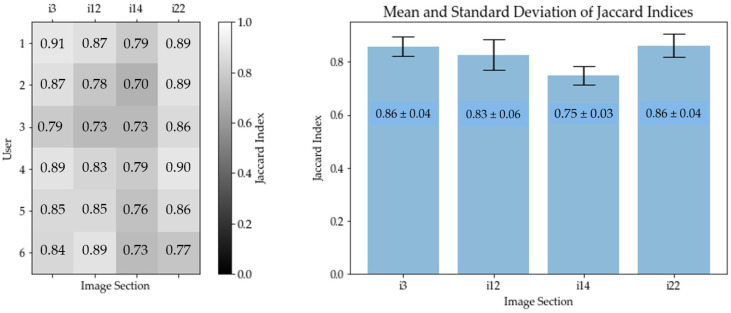
Graphical Jaccard index results of image sections “i3”, “i12”, “i14”, and “i22”. (**Left**): Matrix containing the Jaccard indices for each user and each image section. Each row of the matrix corresponds to a user, while each column corresponds to an image. (**Right**): Mean Jaccard index of the respective image section, including standard deviation.

In this case, the set *M* corresponds to the number of calculated centers of gravity of the MOSES—Impacts algorithm, while *U* corresponds to the number of impacts identified by the user. Table 1 shows the mean values and standard deviations (SDs) of the six users with the number of marked centers of gravity in column 2, as well as the calculated number of centers of gravity, identified by the MOSES—Impacts algorithm, in column 3. The calculated mean Jaccard index [%] and the standard deviations (SDs) are listed in column 5 and illustrated in Figure 12.

In Figure 12 (left), each row of the matrix corresponds to a user, while each column corresponds to an image section. The number indicated in each matrix element is the Jaccard index. A high Jaccard index is indicated by a bright background color, while a low Jaccard index is indicated by a dark background color. In Figure 12 (right), the mean values and standard deviations of the Jaccard indices of all users have been calculated for each image section. The Jaccard index therefore ranges between 75 and 86% (see column 6 of Table 1). The four mean Jaccard indices of the image sections result in a general Jaccard index of (82.0 ± 2.0)%.

As a result of two different impact identification approaches, it has not yet been possible to achieve full agreement in the Jaccard index. The MOSES—Impacts algorithm uses hard contrast boundaries between background and foreground pixels as a basis for detection and separation, while the human user also uses brightness gradients to detect and separate impacts. The ring area of an impact (see Figure 5) still poses challenges for the MOSES—Impact algorithm to accurately recognize an impact. In some cases, the algorithm detects more impacts than present due to an inaccurate contour detection of the impact (see Figure 11). One possible solution to improve the MOSES—Impacts algorithm is to integrate an AI model to further improve the detection and separation of impacts.

### 6.3. Limitations of the Evaluation Study

While defining and evaluating the MOSES—Impacts algorithm, we encountered two limitations, which we want to share to enable future studies based on this work:

Limitation 1: The MOSES—Impacts algorithm has only been evaluated on four image sections; future studies should therefore focus on evaluating the method on a larger set of images.

Limitation 2: Currently, there is no established measure to evaluate detection algorithms in laser–matter experiments. In this paper, the MOSES—Impacts algorithm was compared with human annotations, which we collected ourselves. To be able to make a statement about how well the algorithm performs in comparison to other algorithms, these would have to be evaluated on the same annotations as well.

## 7. Conclusions and Future Work

This paper presents the MOSES—Impacts algorithm to detect and separate connected shrapnel impacts on glass plate surfaces, which occur during laser–matter experiments in a vacuum. Since there was no general definition in the literature describing shrapnel impacts in detail, a definition of a shrapnel impact was worked out and applied in the user study. The input image was filtered with two different morphological operators to recognize and separate connected shrapnel impacts. By merging the information obtained from the two differently filtered images, an optimized result was calculated, which was used to identify the centers of gravity. The MOSES—Impacts algorithm achieved a Jaccard index between 75 and 86% compared to human users.

In addition, the influence of connected impacts, which are automatically separated, was evaluated for a laser recycling process. Separation may save around 7% of the undamaged surface area. This result shows that a separation of impacts in a recycling process is a great advantage. In the future, an AI model could be used to further improve the MOSES—Impacts algorithm, focusing specifically on the ring area.

## Figures and Tables

**Table 1:** Mean Jaccard index results for all image sections evaluated. The first column shows the name of image sections. The second column displays the mean values and corresponding standard deviations SD of identified centers of gravity by the users (*U*). The third column displays the number of identified centers of gravity by the MOSES—Impacts algorithm (*M*). The fourth column shows the mean intersection of centers of gravity, identified by both the users (*U*) and the MOSES—Impacts algorithm (*M*). Finally, the fifth column shows the calculated mean Jaccard index [%].

Image	Mean User Mark (*U*)	Algo. Mark (*M*)	Mean *M* ∩ *U*	Mean Jaccard Index
“i3”	159.6 (SD = 10.7)	165	149.8 (SD = 3.4)	85.86% (SD = 4.07%)
“i12”	118.3 (SD = 15.6)	119	107.2 (SD = 7.0)	82.61% (SD = 6.28%)
“i14”	85.5 (SD = 8.5)	95	77.3 (SD = 4.5)	75.00% (SD = 3.79%)
“i22”	144.0 (SD = 7.7)	129	126.2 (SD = 1.6)	86.14% (SD = 4.78%)

## Data Availability

The data and software underlying the results presented in this paper are not publicly available at this time, but may be obtained from the authors upon reasonable request. All figures presented in the manuscript were created or taken by the first author directly. As such, we hold the original copyrights to these materials and confirm that there are no copyright issues regarding their use in our submission.

## References

[1] Tobin M., Andrew J., Haupt D., Mann K., Poco J., Satcher J., Curran D., Tokheim R., Eder D. (2003). Using Silica Aerogel to Characterize Hypervelocity Shrapnel Produced in High Power Laser Experiments. Int. J. Impact Eng..

[2] Andrew J.E., Edwards R.D., Fyrth J.D., Gardner M.D., Simons A.J., Vaughan K., Allwork C.G., Clarke R.J., Doyle H. (2008). Observations of debris and shrapnel plumes from PW driven solid targets. Cent. Laser Facil. Annu. Rep..

[3] Borneis S., Laštovička T., Sokol M., Jeong T.-M., Condamine F., Renner O., Tikhonchuk V., Bohlin H., Fajstavr A., Hernandez J.-C. (2021). Design, installation and commissioning of the ELI-Beamlines high-power, high-repetition rate HAPLS laser beam transport system to P3. High Pow Laser Science and Engineering.

[4] Booth N., Astbury S., Bryce E., Clarke R.J., Gregory C.D., Green J., Haddock D., Heathcote R.I., Spindloe C. (2018). Debris studies for high-repetition rate and high-power laser experiments at the Central Laser Facility. Radiation Detectors in Medicine, Industry, and National Security XIX.

[5] Bude J., Carr C.W., Miller P.E., Parham T., Whitman P., Monticelli M., Raman R., Cross D., Welday B., Ravizza F. (2017). Particle damage sources for fused silica optics and their mitigation on high energy laser systems. Opt. Express.

[6] Mundhenk T.N., Kegelmeyer L.M., Trummer S.K., Nagahara H., Umeda K., Yamashita A. (2017). Deep learning for evaluating difficult-to-detect incomplete repairs of high fluence laser optics at the National Ignition Facility. Thirteenth International Conference on Quality Control by Artificial Vision 2017, Proceedings of the International Conference on Quality Control by Artificial Vision 2017, Tokyo, Japan, 14 May 2017.

[7] Spaeth M.L., Wegner P.J., Suratwala T.I., Nostrand M.C., Bude J.D., Conder A.D., Folta J.A., Heebner J.E., Kegelmeyer L.M., MacGowan B.J. (2016). Optics Recycle Loop Strategy for NIF Operations above UV Laser-Induced Damage Threshold. Fusion Sci. Technol..

[8] Negres R.A., Abdulla G.M., Cross D.A., Liao Z.M., Carr C.W. (2012). Probability of growth of small damage sites on the exit surface of fused silica optic. Opt. Express.

[9] Peng X., Chen Y., Yu W., Zhou Z., Sun G. (2008). An online defects inspection method for float glass fabrication based on machine vision. Int. J. Adv. Manuf. Technol..

[10] Lebert R., Schriever G., Wilhein T., Niemann B. (1998). Soft X-ray emission of laser-produced plasmas using a low-debris cryogenic nitrogen target. J. Appl. Phys..

[11] Azuma H., Nishimura Y., Sakata A., Takeuchi A. (2002). Debris from tape-target irradiated with pulsed YAG laser. Appl. Surf. Sci..

[12] Higashiguchi T., Rajyaguru C., Dojyo N., Taniguchi Y., Sakita K., Kubodera S., Sasaki W. (2005). Debris characteristics of a laser-produced tin plasma for extreme ultraviolet source. Rev. Sci. Instrum..

[13] Martinkova M., Kalal M., Shmatov M.L. (2013). Analysis of damaging effects of laser-plasma accelerated shrapnels on protecting glass shield. EPJ Web Conf..

[14] Aubert B., Hebert D., Rullier J.-L., Lescoute E., Videau L., Berthe L. (2019). Simulation of laser-driven cratering experiments on aluminum. J. Laser Appl..

[15] Tobin M., Eder D., Braun D., MacGowan B. (2002). Progress on establishing guidelines for National Ignition Facility (NIF) experiments to extend debris shield lifetime. Fusion Eng. Des..

[16] Seisson G., Prudhomme G., Frugier P.-A., Hébert D., Lescoute E., Sollier A., Videau L., Mercier P., Boustie M., Berthe L. (2016). Dynamic fragmentation of graphite under laser-driven shocks: Identification of four damage regimes. Int. J. Impact Eng..

[17] Raman R.N., Negres R.A., Demos S.G. (2011). Kinetics of ejected particles during breakdown in fused silica by nanosecond laser pulses. Appl. Phys. Lett..

[18] Carr C.W. A Brief Review of Fusion Enabling Laser-Induced Damage Reduction, Management, and Repair strategies on at the National Ignition Facility. Proceedings of the Laser-Induced Damage in Optical Materials.

[19] Miller C., Cross D., Senecal J., Clark R., Amorin C., Kegelmeyer L., Carr C.W., Truscott E. (2022). An automated damage inspection microscopy system for National Ignition Facility optics. Laser-Induced Damage in Optical Materials 2022.

[20] Zhao J., Kong Q.-J., Zhao X., Liu J., Liu Y. (2011). A Method for Detection and Classification of Glass Defects in Low Resolution Images. Proceedings of the 2011 Sixth International Conference on Image and Graphics, Graphics (ICIG).

[21] Liu H., Chen Y., Peng X., Xie J. (2011). A classification method of glass defect based on multiresolution and information fusion. Int. J. Adv. Manuf. Technol..

[22] Makaremi M., Razmjooy N., Ramezani M. (2018). A new method for detecting texture defects based on modified local binary pattern. Signal, Image and Video Processing.

[23] Hoang N.-D. (2020). Image Processing-Based Spall Object Detection Using Gabor Filter, Texture Analysis, and Adaptive Moment Estimation (Adam) Optimized Logistic Regression Models. Adv. Civ. Eng..

[24] Otsu N. (1979). A threshold selection method from gray-level histograms. IEEE Transactions on Systems, Man, and Cybernetics.

[25] Cañero-Nieto J.M., Solano-Martos J.F., Martín-Fernández F. (2019). A comparative study of image processing thresholding algorithms on residual oxide scale detection in stainless steel production lines. Procedia Manuf..

[26] Kumar B., Kumar Tiwari U., Kumar S., Tomer V., Kalra J. (2020). Comparison and Performance Evaluation of Boundary Fill and Flood Fill Algorithm. Int. J. Innov. Technol. Explor. Eng..

[27] Burger W., Burge M.J. (2006). Digitale Bildverarbeitung: Eine Einführung mit Java und ImageJ.

[28] Suzuki S., Abe K. (1985). Topological Structural Analysis of Digitized Binary Images by Border Following. Comput. Vis. Graph. Image Process..

[29] Tulsani H., Saxena S., Yadav N. (2013). Segmentation using Morphological Watershed Transformation for Counting Blood Cells. Int. J. Comput. Appl. Inf. Technol..

[30] Ohser J. (2018). Angewandte Bildverarbeitung und Bildanalyse: Methoden, Konzepte und Algorithmen in der Optotechnik, Optischen Messtechnik und Industriellen Qualitätskontrolle.

[31] Beucher S. (1992). The Watershed Transformation Applied to Image Segmentation. Scanning Microsc. Int..

[32] Bieniek A., Moga A. (2000). An efficient watershed algorithm based on connected components. J. Pattern Recognit. Soc..

[33] Cao W., Qiao Z., Gao Z., Lu S., Tian F. (2021). Use of unmanned aerial vehicle imagery and a hybrid algorithm combining a watershed algorithm and adaptive threshold segmentation to extract wheat lodging. Phys. Chem. Earth Parts A/B/C.

